# Aetiology, antimicrobial therapy and outcome of patients with community acquired severe sepsis: a prospective study in a Norwegian university hospital

**DOI:** 10.1186/1471-2334-14-121

**Published:** 2014-03-04

**Authors:** Siri Tandberg Nygård, Nina Langeland, Hans K Flaatten, Rune Fanebust, Oddbjørn Haugen, Steinar Skrede

**Affiliations:** 1 Department of Clinical Science, University of Bergen, Bergen, Norway; 2 Department of Medicine, Haukeland University Hospital, Jonas Lies vei 63, Bergen N 5021, Norway; 3 Department of Anaesthesia and Intensive Care, Haukeland University Hospital, Bergen, Norway; 4 Department of Clinical Medicine, University of Bergen, Bergen, Norway; 5 Department of Heart Diseases, Haukeland University Hospital, Bergen, Norway

**Keywords:** Severe sepsis, Epidemiology, Aetiology, Antimicrobial therapy, Compliance, Outcome

## Abstract

**Background:**

Severe sepsis is recognized as an inflammatory response causing organ dysfunction in patients with infection. Antimicrobial therapy is the mainstay of treatment. There is an ongoing demand for local surveillance of sepsis aetiology and monitoring of empirical treatment recommendations. The present study was established to describe the characteristics, quality of handling and outcome of patients with severe sepsis admitted to a Norwegian university hospital.

**Methods:**

A one year prospective, observational study of adult community acquired case-defined severe sepsis was undertaken. Demographics, focus of infection, microbiological findings, timing and adequacy of empirical antimicrobial agents were recorded. Clinical diagnostic practice was evaluated. Differences between categorical groups were analysed with Pearson’s chi-squared test. Predictors of in-hospital mortality were identified in a multivariate stepwise backward logistic regression model.

**Results:**

In total 220 patients were identified, yielding an estimated annual incidence of 0.5/1000 inhabitants. The focus of infection was established at admission in 69%. Respiratory tract infection was present in 52%, while genitourinary, soft tissue and abdominal infections each were found in 12-14%. Microbiological aetiology was identified in 61%; most prevalent were *Streptococcus pneumoniae*, *Escherichia coli* and *Staphylococcus aureus*. Independent predictors of in-hospital mortality were malignancy, cardiovascular disease, endocarditis, abdominal infections, undefined microbiological aetiology, delay in administration of empirical antimicrobial agents ≥ 6 hours and use of inadequate antimicrobial agents. In patients ≥ 75 years, antimicrobial therapy was less in compliance with current recommendations and more delayed.

**Conclusions:**

Community acquired severe sepsis is common. Initial clinical aetiology is often revised. Compliance with recommendations for empirical antimicrobial treatment is lowest in elderly patients. Our results emphasizes that quick identification of correct source of infection, proper sampling for microbiological analyses, and fast administration of adequate antimicrobial agents are crucial points in the management of severe sepsis.

## Background

Sepsis is recognized as a dysregulation of the inflammatory response in patients with infection. Progression to severe forms with organ dysfunction develops in one out of three patients, commonly resulting in long-term hospitalization and death [1,2]. Algorithms for identification and management of severe infection hence emphasize recordings of vital signs and laboratory data, aiming at discovering circulatory failure and other organ dysfunctions at an early stage [3,4]. In recent years, one of the main focuses in sepsis related research has been on candidate biomarkers in the host and their possible role in targeted therapy. However, definite novel therapeutic approaches have not been established and optimized anti-infective therapy is still the mainstay of treatment in severe sepsis. Delayed administration of the initial dose of antimicrobial agents, the adequacy of antimicrobial therapy and, where applicable, delayed surgical source control, are all independent prognostic factors [5-8]. Care bundles with guidance on how to diagnose and handle affected patients, including up-to-date recommendations on empirical treatment, therefore emerges as one of the most important measures to improve outcomes in sepsis. In 2007, Llewelyn and Cohen addressed the relevance of monitoring the microbial epidemiology of sepsis on a local basis [9]. Based on such knowledge, empirical recommendations may be customized. Accordingly, we established the present study of adult community acquired severe sepsis to describe the occurrence, characteristics and handling of affected patients admitted to our hospital. Secondary objectives were to evaluate our physicians’ compliance with local guidelines and to identify potential predictors of in-hospital mortality in severe sepsis.

## Methods

### Study setting

Haukeland University Hospital is serving as a local hospital with approximately 350.000 inhabitants in the catchment area. It is also a tertiary care referral center in western Norway, with a population of 1.1 million inhabitants. The current investigation was a one year prospective, case defined observational study of patients hospitalized in the period from January 1st through December 2008. Enrollment of patients took place in the high dependency unit at the Division for infectious diseases, Department of Medicine, in the general intensive care unit (ICU) at the Department of Anesthesia and Intensive care, and in the combined intensive care and high dependency unit at the Department of Cardiology.

### Patient selection

All subjects transferred from the emergency department (ED) to any of the three units were screened for severe sepsis according to consensus criteria [3,4]. Patients older than 15 years of age hospitalized due to community acquired infection, including patients transferred from affiliated hospitals, were included if they developed severe sepsis within 24 hours of admission to the primary institution. Five patients were not recognized within 24 hours, but suspected to have non-infectious conditions. However, they were identified with delay and included, as their fulfillment of inclusion criteria within the first 24 hours of hospitalization was documented. Daily screening in the three units involved were performed. Patients were evaluated for eligibility by use of admission records, patient charts and inquiries with senior physicians at the respective wards. All of the eligible subjects were discussed in consensus meetings within the group of co-authors before a final decision of inclusion was made.

### Data collection and follow-up

Clinical data were registered prospectively until hospital discharge or in-hospital death, using predefined case report forms. Information was collected from medical records, patient charts, and the intensive care electronic monitoring system IntelliVue Clinical Information Portfolio (ICIP, Philips Medical Systems, Eindhoven, the Netherlands). The following parameters were recorded at admission; time and date, department affiliation, demographics, comorbidities, suspected focus of infection, heart rate, respiratory rate, blood pressure, body temperature and Glasgow coma scale (GCS). Laboratory results were registered continuously. During the follow-up, data on organ dysfunction and adjunctive sepsis therapies was recorded. Timing and adequacy of antimicrobial agents was evaluated together with their appropriateness according to local recommendations. Results from blood cultures and microbiological analyses of urine, abscess drainage, sputum, feces and cerebrospinal fluid were collected. Possible contaminants of samples were excluded from analysis. Antimicrobial susceptibility patterns of cultured pathogens were registered when available. At discharge a final diagnosis was established by one consultant (SS), based on retrospective evaluation of all available records, clinical, microbiological, laboratory and medical imaging data. Data from medical records and patient charts was verified before it was entered in a local database. The main outcome measure was the in-hospital case fatality rate (CFR). We also calculated the 28-day all-cause mortality rate. Long-term survival was assessed after four years.

### Calculation of incidence and mortality rate

The population incidence and the 28-day all-cause mortality rate was calculated based on the number of inhabitants > 15 years in the local catchment area in the year 2008. Patients transferred from affiliated hospitals were excluded from this estimation. Incidence per hospital admissions was calculated from total cases in the study.

### Statistical analyses

Data were analyzed using PASW Statistics 18 software (SPSS Inc., Chicago, Ill. USA). Differences between categorical groups were analyzed with Pearson’s chi-squared test (χ^2^). To identify predictors of outcome, univariate logistic regression analyses of factors previously reported to be associated with mortality was performed. Variables with P values < .10 were entered into a multivariate stepwise backward model. Results from the logistic regression analyses are reported as odds ratios (ORs) with 95% confidence intervals (CIs) from the unadjusted (univariate) models, the fully adjusted model (step 1 in backward stepwise regression), and final model (4th and last step in backward stepwise regression) with p-values from the likelihood ratio test. For the latter two models the results of Hosmer-Lemeshow’s goodness-of-fit-test are reported. All variables in the logistic regression analyses presented in table form are categorical. We additionally tested for linear trend across age groups with 15 years intervals, with time to administration of the initial antimicrobial agents assessed as a continuous variable measured in hours (with exclusion of two extreme outlying values > 100 hours) and with time assessed as a continuous variable after logarithmic transformation. Long-term survival was compared across age groups by a Kaplan-Meier plot with log rank computation results. Overall, two sided P values < 0.05 were considered significant.

### Ethical considerations

The study was approved by the Regional Committee for Medical and Health Research Ethics, Health Region West (REK-Vest, case number 2010/165).

## Results

### Base-line characteristics

A total of 220 patients with community acquired severe sepsis were identified, corresponding to an annual incidence of 2.2/1000 hospital admissions and 0.5/1000 inhabitants. Median age was 67 years and there was a small predominance of male patients (53%). Significant comorbidity was present in 90%.

### Focus of infection

A final clinical diagnosis was established in all patients at discharge (Table 1). At admission, the correct primary focus of infection was identified in 69%. The focus was considered unidentified in 16%, an incorrect focus was suggested in 11%, whereas the remaining 4% were not suspected to have infection at admission.

**Table 1:** Suspected, confirmed and proportion of correct identified focus of infection in community acquired severe sepsis (n (%))

**Infection**	**Suspected at admission**^**a**^	**Confirmed at discharge**^**a**^	**Correct at admission**^**b**^
Respiratory	101 (45.9)	115 (52.3)	93 (80.9)
Genitourinary	25 (11.4)	31 (14.1)	20 (64.5)
Soft tissue	23 (10.5)	27 (12.3)	18 (66.7)
Abdominal	16 (7.3)	26 (11.8)	13 (50)
Endocarditis	4 (1.8)	12 (5.5)	4 (33.3)
Bacteremia	2 (0.9)	5 (2.3)	1 [20]
CNS	4 (1.8)	4 (1.8)	2 (50)
Unknown	36 (16.4)	0 (0.0)	n.a.
Not suspected	9 (4.1)	n.a.	n.a.
Total	220 (100)	220 (100)	151 (68.6)

Abbreviations: CNS, central nervous system; n.a., not applicable.

^a^Percent calculated column-wise, from total cases.

^b^Percent calculated row-wise, from each infection category’s total number of confirmed cases.

The level of diagnostic precision differed depending on the nature of the infection. Respiratory tract infection (RTI) was e.g. suspected in 101 subjects at admission, of whom 93 were later confirmed while 8 turned out to have another focus. Overall, RTI was verified in 115 patients and among these, 22 cases were missed at admission. As follows, 81% of actual RTIs were assigned with a correct diagnosis in the ED. In the less frequent causes of sepsis the level of precision was lower (Table 1).

### Microbiology

**Figure 1:**
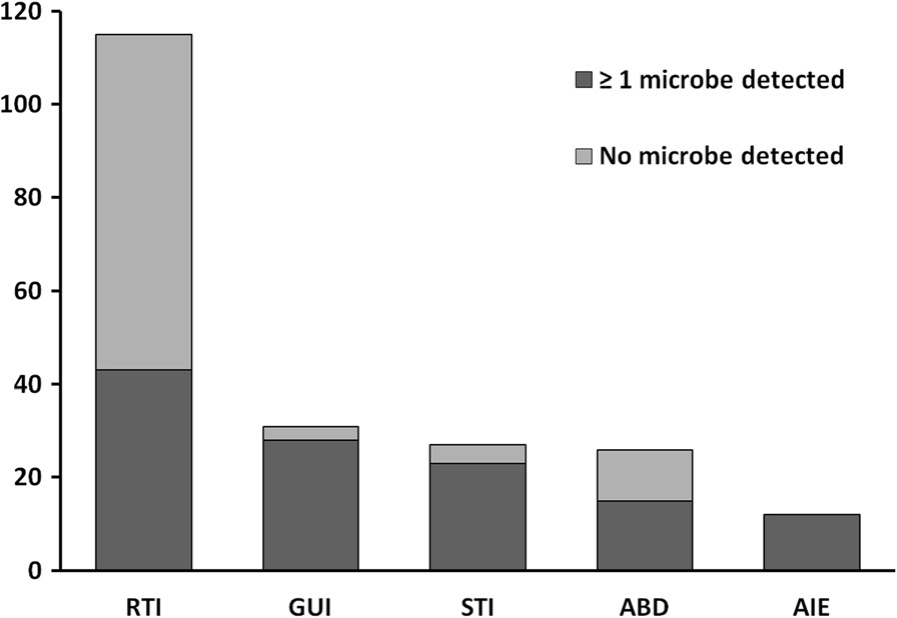
**Microbiological identification rates in different infection categories (n).** Relationship between focus of infection and the proportion of patients with confirmed microbiological aetiology. The most prevalent microbe was in RTI (respiratory tract infection); *Streptococcus pneumoniae* (28/43), GUI (genitourinary infection); *Escherichia coli* (16/28), STI (soft tissue infection); Group A/C/G streptococci (10/23), ABD (abdominal infection); *Escherichia coli* (8/15), and AIE (acute infectious endocarditis); *Staphylococcus aureus* (5/12).

Microbiological tests were performed in 212 of 220 patients. A plausible pathogen was identified in 61% of tested subjects, with a total of 171 positive tests all together (Table 2). Gram-positive microbes constituted 57%. Overall, *Streptococcus pneumoniae* was most prevalent, closely followed by *Escherichia coli*, *Staphylococcus aureus* and alpha hemolytic streptococci. Blood cultures were obtained in 198 cases. Of these, 37% were positive, most often with *E. coli*, *S. pneumoniae* or *S. aureus* respectively. Among the most prevalent bacteria, resistance towards empirical antimicrobial regimens was observed in two isolates only. Both were ESBL-producing *E. coli*. Polymicrobial infections were found in 19% of test-positive patients, namely in eight soft tissue infections (STIs), six abdominal infections, six RTIs (aspiration in all cases) and four genitourinary infections (GUIs). Figure 1 shows the relationship between the focus of infection and the proportion of patients with confirmed microbiological aetiology. Fifteen subjects received antimicrobial treatment prior to hospitalization. Microbiological samples were obtained from all of them, yet a plausible pathogen was identified in only five cases.

**Table 2:** Microbiological aetiology in community acquired severe sepsis (n)

	**Total**	**Blood**	**Urine**	**Abscess drainage**	**Other**
**Gram-positive**^**a**^	90	44	27	25	18
*Streptococcus pneumonia*	29	14	20^b^	0	5
*Alpha hemolytic streptococci*	18	7	0	6	7
*Group A/C/G streptococci*	13	6	0	9	1
*Group B streptococci*	2	1	0	1	0
*Enterococci*	6	3	2	0	2
*Staphylococcus aureus*	20	11	4	9	3
*Staphylococcus caprae*	1	1	0	0	0
*Aerococcus viridans*	1	1	1	0	0
**Gram-negative**^**a**^	55	32	21	8	9
*Escherichia coli*	27	19	13	3	3
*Klebsiella*	10	6	5	0	1
*Enterobacter*	1	1	0	0	0
*Proteus*	2	0	0	1	1
*Other Enterobacteriaceae*	5	2	3	1	0
*Pseudomonas aeruginosa*	2	1	0	1	1
*Stenotrophomonas maltophilia*	1	0	0	1	0
*Neisseriae meningitides*	2	2	0	0	0
*Haemophilus influenzae*	2	1	0	0	1
*Haemophilus parainfluenzae*	2	0	0	0	2
*Unspecified gram negative rods*	1	0	0	1	0
**Anaerobic bacteria**	17	6	0	3	9
*Clostridium species*	5	2	0	0	3^c^
*Bacteroides species*	5	3	0	1	2
*Prevotella*	4	0	0	2	2
*Slackia exigua*	1	1	0	0	0
*Fusobacterium*	1	0	0	0	1
*Unspecified gram positive rods*	1	0	0	0	1
**Other**	9	0	0	3	6
*Candida species*	7	0	0	3	4
*Aspergillus species*	1	0	0	0	1
*Influenzavirus A*	1	0	0	0	1
**Patients with ≥1 positive test**	129	74	40	23	29

Unless otherwise specified, numbers shown are all isolated microorganisms in category.

^a^Anaerobic species not included.

^b^Positive antigen tests in all 20 cases (14 cases were detected in antigen tests only).

^c^Detection of *Clostridium difficile* toxin A in all cases.

### Antimicrobial agents

Table 3 outlines the initial choice of empirical antimicrobial agents and the physicians’ compliance with the recommendations for empirical treatment of severe sepsis at our hospital in 2008. Considering the suspected focus of infection at admission, the empirical choice of antimicrobial therapy was correct in 81% of the cases. Compliance with the recommendations was lower when patients were ≥ 75 years of age (*χ*^2^ P = 0.029). When comparing the initial given agent with the confirmed focus at discharge, 76% had received empirical treatment appropriate for their final diagnosis. Susceptibility tests revealed that in the group with defined microbiological aetiology (n = 129), 82% had been treated with adequate antimicrobial therapy from the first dose. Compliance with the recommendations proved to have been better when adequate therapy was given, in comparison to when inadequate therapy was given (83% vs. 44%, *χ*^2^ P < 0.001).

**Table 3:** Choice of empirical antimicrobial regimen according to suspected and confirmed focus of infection and compliance with recommendations (n/n (%))

**Focus of infection with recommended regimen**	**Suspected focus at admission Total cases/Cases with correct regimen**^**a**^	**Confirmed focus at discharge Total cases/Cases with appropriate regimen**^**a**^
**Respiratory**^**b,c**^	100^d^/82 (82.0)	115^d^/96 (83.5)
*penicillin G* and *ciprofloxacin* or		
*penicillin G* and *gentamicin*^e,f^		
**Genitourinary**	25^d^/20 (80.0)	30^d^/24 (80.0)
*ampicillin* and *gentamicin*^f^		
**Soft tissue**	23/18 (78.3)	27^d^/18 (66.7)
*penicillin G* and *clindamycin* (+/- *gentamicin*)		
**Abdominal**	16^d^/11 (68.8)	26^d^/13 (50.0)
*ampicillin* and *gentamicin* and *metronidazol* or		
*3rd generation cephalosporin* and *metronidazol* or		
*piperacillin-tazobactam* or		
*meropenem*		
**Endocarditis**	4/3 (75.0)	12/7 (58.3)
*penicillin G* and *gentamicin* or		
*3rd generation cephalosporin*		
**CNS**	4/3 (75.0)	4/4 (100)
*penicillin G* and *3rd generation cephalosporin*		
**Unknown/bacteremia**	38^d^/34 (89.5)	5/5 (100)
*penicillin G* and *gentamicin* (+/- *metronidazol*)^e,f^		
**Total**	210/171 (81.4)	219/167 (76.3)

^a^Correct and appropriate regimen according to recommendations for empirical antimicrobial therapy in Haukeland University Hospital in 2008.

^b^One patient with a suspected and later verified respiratory tract infection died before antimicrobial therapy was implemented.

^c^Suspected atypical pneumonia: macrolide or doxycycline is added.

^d^Number of correct cases including one patient given meropenem as initial agent (n = 4 in total).

^e^If allergic to penicillin: clindamycin.

^f^If gentamicin is contraindicated: 3rd generation cephalosporin monotherapy.

Median delay before administration of the initial dose of antimicrobial therapy was 2.8 hours after hospital admission. Compared to subjects with RTI, the delay was highest in abdominal infections (2.5 hours vs. 6.9 hours). Patients in the latter category had a 4.5 times greater risk of receiving their initial dose after more than six hours of hospitalization (95% CI 1.8-11.0, *χ*^2^ P = 0.005). Moreover, patients ≥ 75 years of age had a 2.3 times greater risk of receiving antimicrobial therapy beyond six hours compared to younger patients (95% CI 1.2- 4.4, *χ*^2^ P = 0.008). Among cases with no suspicion of infection at admission, the delay was 13 – 75 hours.

### Outcome

**Figure 2:**
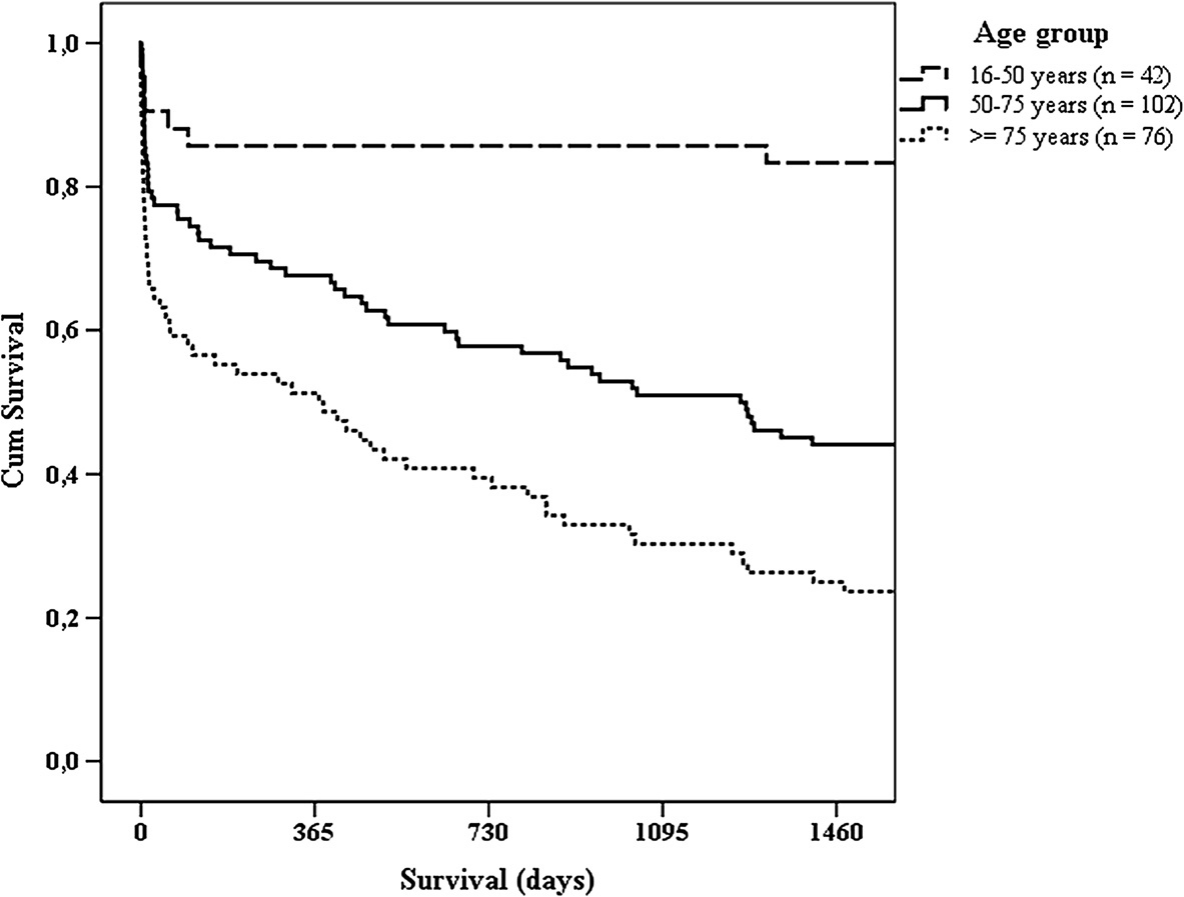
**Long-term survival after community acquired severe sepsis.** Kaplan-Meier curve on survival after community acquired severe sepsis in different age groups. Follow-up was four years after hospital admission for all 220 patients. Survival in all three groups were significant different according to Log rank test results (P = 0.000 in analysis of age group 16–50 vs. 50–75 and P = 0.001 in analysis of age group 50–75 vs. ≥75). Overall, four-year mortality was 55.5%.

Table 4 shows case fatality rates (CFRs) for subgroups of patients and predictors of outcome in uni- and multivariate logistic regression analyses. High CFRs were seen in patients with malignancy or cardiovascular disease. In the multivariate analysis, patients with confirmed endocarditis and abdominal infections had a significantly greater risk of death compared to RTI. Detection of microbiological aetiology and adequate antimicrobial treatment increased survival. Mortality was increased when antimicrobial agents had been given before hospital admission and when time to administration of the initial in-hospital dose of antimicrobial agents was more than six hours. There were not significant results in multivariate analyses with cut-offs below six hours or when testing for linear trend in mortality according to hourly increasing delays of antimicrobial agents. When limiting the latter analysis to patients receiving appropriate empirical therapy (adequate antimicrobial agents in cases with detection of a plausible pathogen, and correct empirical antimicrobial agents according to Hospital guidelines in patients with no detection of a pathogen, n = 178) there was a small significant impact also in multivariate analysis (p = 0.001, OR 1.06, 95% CI: 1.02 to 1.09). In univariate analyses, patients with a correctly identified source of infection at admission and patients receiving appropriate empirical antimicrobial agents according to Hospital guidelines had a higher chance of survival. However, these findings could not be validated by multivariate analysis. A tendency towards greater mortality with increasing age was observed, but was not statistically significant. Replacing age categories in Table 4 with trend across age groups provided a similar non-significant result in multivariate analysis, as did categorization of patients into two groups on the basis of age below versus ≥75 years. Total in-hospital CFR in this study was 25%. The 28-day CFR was 24.5% and the 28-day all-cause mortality rate was 13/100 000 inhabitants per year. One-year mortality was 34.5%. A Kaplan-Meier curve on survival is presented in Figure 2.

**Table 4:** In-hospital mortality in patients with community acquired severe sepsis at Haukeland University Hospital in 2008

	**All**^**a**^	**Non-survivors**		**Unadjusted models**			**Fully adjusted model**^**b**^			**Final model**^**c**^		
**Characteristic**	**n**	**n**	**(%)**	**OR**	**95% CI**	**P value**	**OR**	**95% CI**	**P value**	**OR**	**95% CI**	**P value**
Gender						0.240						
Male	117	33	(28.2)	1.00	Reference							
Female	103	22	(21.4)	0.69	(0.37, 1.29)							
Age (years)						0.065			0.619			
16-30	18	1	(5.6)	1.00	Reference		1.00	Reference				
30-45	22	4	(18.2)	3.78	(0.38, 37.28)		0.99	(0.07, 14.18)				
45-60	36	8	(22.2)	4.86	(0.56, 42.30)		2.27	(0.19, 26.64)				
60-75	68	16	(23.5)	5.23	(0.65, 42.43)		2.88	(0.28, 29.54)				
≥75	76	26	(34.2)	8.84	(1.11, 70.18)		3.09	(0.28, 33.54)				
Comorbidity												
None	23	1	(4.3)	0.12	(0.02, 0.92)	0.005						
Hypertension	91	18	(19.8)	0.61	(0.32, 1.17)	0.130						
Cardiovascular	107	35	(32.7)	2.26	(1.20, 4.24)	0.010	2.18	(0.85, 5.61)	0.101	3.29	(1.45, 7.48)	0.003
Pulmonary	61	14	(23.0)	0.86	(0.43, 1.72)	0.662						
Diabetes	38	9	(23.7)	0.92	(0.40, 2.08)	0.836						
Malignancy	31	13	(41.9)	2.53	(1.15, 5.58)	0.025	5.97	(1.96, 18.19)	0.001	5.50	(1.92, 15.78)	0.001
Dementia	17	6	(35.3)	1.71	(0.60, 4.88)	0.324						
Psychiatric	51	9	(17.6)	0.57	(0.26, 1.27)	0.155						
Substance abuse	31	6	(19.4)	0.69	(0.27, 1.77)	0.423						
Other^d^	74	27	(36.5)	2.42	(1.29, 4.53)	0.006	2.52	(1.11, 5.70)	0.025	2.43	(1.10, 5.35)	0.026
Correct suspected focus of infection						0.020			0.606			
Yes	152	31	(20.4)	1.00	Reference		1.00	Reference				
No	68	24	(35.3)	2.13	(1.13, 4.02)		0.79	(0.33, 1.91)				
Confirmed focus of infection						0.007			0.003			0.001
Respiratory	115	25	(21.7)	1.00	Reference		1.00	Reference				
Genitourinary	31	4	(12.9)	0.53	(0.17, 1.67)		0.41	(0.08, 2.21)		0.47	(0.09, 2.39)	
Soft tissue	27	6	(22.2)	1.03	(0.38, 2.82)		2.04	(0.55, 7.60)		2.42	(0.68, 8.68)	
Abdominal	26	12	(46.2)	3.09	(1.27, 7.51)		2.95	(0.87, 10.03)		3.54	(1.09, 11.43)	
Endocarditis	12	7	(58.3)	5.04	(1.47, 17.25)		17.43	(2.74, 111.06)		18.94	(3.45, 104.06)	
Bacteremia	5	0	(0.0)	0.00	(0.00, )		0.00	(0.00, )		0.00	(0.00, )	
CNS	4	1	(25.0)	1.20	(0.12, 12.04)		9.22	(0.71, 118.97)		7.66	(0.63, 93.73)	
Microbiological samples						0.008			0.028			0.025
Positive	129	24	(18.6)	1.00	Reference		1.00	Reference		1.00	Reference	
Negative	83	26	(31.3)	2.00	(1.05, 3.79)		3.58	(1.34, 9.55)		3.34	(1.29, 8.63)	
Not obtained	8	5	(62.5)	7.29	(1.63, 32.63)		2.91	(0.35, 24.09)		4.44	(0.60, 32.95)	
Empirical antimicrobial agents												
Suspected focus of infection						0.433						
*Appropriate compliance*	171	38	(22.2)	1.00	Reference							
*Inappropriate compliance*	39	11	(28.2)	1.38	(0.63, 3.02)							
Confirmed focus of infection						0.027			0.241			
*Appropriate*	168	35	(21.0)	1.00	Reference		1.00	Reference				
*Inappropriate*	52	19	(36.5)	2.17	(1.10, 4.27)		0.79	(0.33, 1.91)				
Microbiological aetiology^e^						< 0.001						
*Adequate*	106	12	(11.3)	1.00	Reference							
*Inadequate*	23	12	(52.2)	8.55	(3.10, 23.58)							
In-hospital initial dose administered						0.002			0.051			0.046
*<6 hours after admission*	157	30	(19.1)	1.00	Reference		1.00	Reference		1.00	Reference	
≥*6 hours after admission*	54	22	(40.7)	2.91	(1.49, 5.71)		2.52	(1.00, 6.38)		2.48	(1.02, 6.02)	
Pre-hospital administration						0.059			0.055			0.041
*No*	205	48	(23.4)	1.00	Reference		1.00	Reference		1.00	Reference	
*Yes*	15	7	(46.7)	2.86	(0.99, 8.30)		4.13	(0.99, 17.21)		4.20	(1.08, 16.39)	

*Abbreviations:* OR: odds ratio; CI: confidence interval.

^a^n = 220.

^b^Includes all categories with P < 0.10 in the unadjusted analyses; n = 211; Hosmer-Lemeshow’s chi-square = 12.38, df = 8, P = 0.135.

^c^From backward stepwise selection at significance level 0.05; n = 211; Hosmer-Lemeshow’s chi-square = 3.18, df = 8, P = 0.923.

^d^Including chronic kidney, liver and rheumatic diseases.

^e^Not included in multivariate analysis due to a substantial number of not applicable cases (if included, significant in multivariate analysis).

## Discussion

We estimated the annual incidence of community acquired severe sepsis in our hospital to be 2.2/1000 admissions and 0.5/1000 inhabitants. In-hospital mortality was 25% and the 28-day all-cause mortality rate was 13/100 000 inhabitants per year.

Previous data on the occurrence of sepsis in Norway is limited to a retrospective study using data from the Norwegian Patient Registry, in which the total incidence of severe sepsis was calculated to 3.0/1000 admissions and 0.47/1000 inhabitants [10]. In contrast to this study, we did not include nosocomial sepsis which constitutes approximately 50% of all cases [5,6,11-14]. During three months prior to the present study we performed a prospective pilot survey of case-defined sepsis in our ED. Subsequent comparison with cases identified retrospectively by discharge ICD-10 codes revealed that 50% of all sepsis-cases were missed when using the retrospective method alone (Nygård, unpublished results). Thus, the calculation in our current prospective study probably represents a more accurate estimation of the Norwegian incidence of community acquired severe sepsis than previous results. Previous prospective studies with systematic inclusion from both ICUs and non-ICUs, reporting details on the occurrence of severe sepsis, are to our knowledge limited to a single survey from Spain [15]. They calculated an annual incidence of 1.0/1000 inhabitants, including nosocomial cases. Other studies of community acquired severe sepsis have used different methods for patient inclusion, and many do not offer data on incidence or are not applicable for such purpose [16-22].

The respiratory tract was the most frequent origin of infection in our patients. This is consistent with results from other studies [5,11-14,23-25]. We diagnosed abdominal infections less frequently, while GUI and STI were found more often than in many previous reports [6,11-13,25-27]. However, studies with inclusion of patients treated outside ICUs have found a distribution of diagnoses more similar to ours [15,16,23,28,29]. Patients with abdominal infections have been shown to have a high demand for intensive care treatment [11]. There are in addition more abdominal infections in nosocomial compared to community acquired sepsis [15,30]. In our study most patients with this diagnosis were treated in an ICU, whereas patients with GUI on the contrary mainly were treated outside ICUs. Moreover, patients with abdominal infection had a prolonged length of stay (data not shown). In many studies on sepsis epidemiology, frequencies are estimated on the basis of prevalence data from ICUs only. Together, these observations suggest that distribution of various infections is influenced by study design, e.g. our low occurrence of abdominal infections is likely conditional to inclusion of patients from outside ICUs.

A likely pathogen has been found in 60-75% of eligible subjects in other studies on sepsis [5,6,13,19,24,31], comprising positive blood cultures within the range of 22-37% [5,6,12,19,30-33]. This is comparable to our data. In line with related observations, we identified more Gram-positive than Gram-negative bacteria [1,12,24]. Antigen- and toxin tests were included in our analysis of microbiological aetiology. A positive urinary antigen assay was the only laboratory documentation in 48% of subjects with *S. pneumoniae*. This is in accordance with a prospective report on community acquired pneumonia, where 44% of 171 cases with *S. pneumoniae* were diagnosed by urinary antigen detection alone [34]. In Norway, guidelines for pneumococcal vaccination of adults ≥ 65 years and pre-defined younger persons at risk have been established since 1996, but vaccine coverage has not been satisfactory. However, a pneumococcal conjugated vaccine has been a part of the routine vaccination program for children with nearly completely coverage since 2006. From this point there has been a marked decline in the number of cases with invasive *S. pneumoniae*, also among adults. Thus, the proportion of patients with severe sepsis originating from this microbe is probably decreasing.

The proportion of patients with detection of a plausible pathogen differed among the various origins of infection in our study. The detection rate was low in RTI and abdominal infection in particular. This is also previously documented [20,31]. Patients with negative microbiological samples had a significant greater risk of death than patients with positive samples. Although not significant in all categories, this result is consistent when stratification of infection categories is performed, supporting that the increased risk of death in cases with negative samples was retained throughout our multivariate model. Detection of a plausible pathogen gives the opportunity to administer validated adequate antimicrobial treatment. In subjects with negative microbiological samples, correct antimicrobial therapy can be delayed or not provided. An increased risk of death has been demonstrated in patients with severe infection receiving inadequate antimicrobial therapy [6,7,35-37]. In many of these studies multiresistant isolates were often encountered. We identified no isolates of MRSA, MRSE or VRE, and only two cases of ESBL-producing Gram-negative bacilli (of which one was even suspected, and treated accordingly, at admission). On the contrary, *S. pneumoniae* and *E. coli* were frequently identified. The CFRs in these two categories were low; 7 and 18.5% respectively (data not shown). Hence, a possible contributor to the different risk of death among patients with positive versus negative microbiological samples might be our high frequency of microbes with unproblematic resistance properties. Inability to tailor treatment with effective antimicrobial agents might also have influenced outcomes of the patients which received pre-hospital antimicrobial agents. In a study by Garnacho-Montero et al., previous antibiotic therapy within the last month was independently related to administration of inadequate antimicrobial therapy [6].

We were able to demonstrate a correlation between correct use of empirical antimicrobial agents and susceptibility in detected pathogens. It is therefore of concern that one in five patients did not receive the recommended regimen. One possible explanation for this is that sepsis is diagnosed on the basis of unspecific criteria, independent of primary focus, microbiological aetiology and host factors. Current diagnostic algorithms focus on identifying complications of an infection rather than its aetiology. The level of precision in establishing the focus of infection was variable and often low in our study. Only 17 of 38 patients with confirmed abdominal infection or endocarditis were assigned with the correct focus, and half of them received initial treatment as recommended. The longest delays before initiation of antimicrobial therapy were found in these two groups, and their hospital mortality was high. An evaluation of the quality of clinical diagnostic practice in severe sepsis, comparing the suspected focus at admission with a confirmed diagnosis at discharge, has to our knowledge not been published previously. We identified severe sepsis in nine subjects not suspected with infection at admission. Opposite, a study of the aetiology of illness in suspected severe sepsis found that 18% of the patients had noninfectious diagnoses mimicking sepsis [20]. Seen together these observations suggest a two-sided limitation in commonly used sepsis algorithms.

We found that a six hour delay or more in administration of the initial dose of antimicrobial treatment was associated with an increased risk of death, but could not demonstrate independent impact on mortality during the preceding hours, as reported by Kumar et al. in a study of patients with septic shock [5]. Other studies investigating the impact of early administration of antimicrobial therapy have also failed to demonstrate an hourly decrease in survival [36,38]. Some have demonstrated beneficial effects on survival with administration of antimicrobial therapy within the first hour [36,39]. Kumar et al. limited their inclusion to cases given effective antimicrobial therapy. Likewise, the beneficial effect found by Gaieski et al. was significant only when antimicrobial therapy was considered appropriate [36]. Analyses of our data with the same limitations as Kumar and Gaieski resulted in significant impact of hourly increasing time to administration of antimicrobial agents on mortality in multivariate analysis. However, the hourly effect was low. Since we have included a broad selection of the population with severe sepsis, ranging from septic shock to cases with other and less severe organ dysfunctions, our results concerning timing is inevitably influenced by the different levels of severity in our population. We were not able to severity stratify our patients and cannot investigate this matter any further.

In patients ≥ 75 years, antimicrobial therapy was less in compliance with current recommendations and more delayed. An age dependent risk of in-hospital mortality has been demonstrated in severe infection [40]. Following the results in our study, we question whether this is solely caused by host factors. Increasing age did not emerge as an independent risk factor. Subjects ≥ 75 years had on average one additional comorbidity, a significant higher presence of cognitive impairment and a significant higher creatinine level at admission than younger patients (data not shown). These data indicates that there were more potentially complicating factors among elderly patients. However, our Hospital guidelines are clear in terms of instructions on adjusting the doses of antimicrobial agents when needed, as well as recommending alternative treatment if the primary choice of drug is contraindicated. This was taken into account when we evaluated the level of compliance. Hence, we consider that there is room for improvements in the handling of our elderly patients, especially given the small difference in mortality after hospital discharge between patients aged 50–75 versus ≥75 years during the long-term follow-up (Figure 2).

### Strengths and limitations

Major strengths of this study are the prospective design, inclusion throughout an entire year, a small group of dedicated investigators and recruitment of patients from both ICUs and non-ICUs. Major limitations are the sample size and a lack of severity stratification of the included patients. Consequently, statistical results are encumbered with uncertainties. Due to the high number of explanatory variables included in the logistic regression analysis and the screening and stepwise selection of variables in the reported results, over fitting is most likely present in the final model reported. As no formal adjustment for multiple testing has been performed, most emphasize should thus be given to the most significant predictors (i.e. with P < 0.01).

## Conclusion

We have found a high incidence of community acquired severe sepsis in a Norwegian university hospital. Initial clinical aetiology was often revised and the diagnosis sometimes overlooked in the emergency department. Adequate antimicrobial therapy improved outcome, while undefined microbiological aetiology, endocarditis, abdominal infections and delayed administration of antimicrobial agents increased the risk of death. A need for improved handling of elderly patients was identified. Our results emphasizes that quick identification of correct source of infection, proper sampling for microbiological analyses, and fast administration of adequate antimicrobial agents are crucial points in the management of severe sepsis.

## Abbreviations

ABD: Abdominal infection; AIE: Acute infectious endocarditis; CI: Confidence interval; CFR: Case fatality rate; ED: Emergency department; ESBL: Extended-spectrum beta-lactamase; GCS: Glasgow coma scale; GUI: Genitourinary infection; ICU: Intensive care unit; OR: Odds ratio; RTI: Respiratory tract infection; STI: Soft tissue infection.

## Competing interests

The study has been financed by the Department of Medicine, Haukeland University Hospital. All the authors report no competing interests.

## Authors’ contributions

STN participated in the design of the study, inclusion of cases, data collection, statistical analyses and drafted the manuscript. NL participated in the design of the study, inclusion of cases, statistical analyses and helped to draft the manuscript. HF participated in inclusion of cases, statistical analyses and drafting of the manuscript. RF and OH participated in inclusion of cases and drafting of the manuscript. SS participated in all parts of the present work. All the authors contributed to and approved the final manuscript.

## Pre-publication history

The pre-publication history for this paper can be accessed here:

http://www.biomedcentral.com/1471-2334/14/121/prepub
